# Supplementary material for: VPS13B is localized at the cis-trans Golgi complex interface and is a functional partner of FAM177A1
Source: bioRxiv. 2023 Dec 18:2023.12.18.572081. Preprint. [Version 1] doi: 10.1101/2023.12.18.572081 (PMC10769246; doi:10.1101/2023.12.18.572081)
Supplement: 1 [file NIHPP2023.12.18.572081V1-supplement-1.pdf]

bioRxiv preprint doi: https://doi.org/10.1101/2023.12.18.572081; this version posted December 18, 2023. The copyright holder for this preprint (which was not certified by peer review) is the author/funder, who has granted bioRxiv a license to display the preprint in perpetuity. It is made available under aCC-BY 4.0 International license.

Supplemental Figure Legends

Supplemental Figure 1. Structure and localization of VPS13B

(A) Domain structure of human VPS13B. **(B)** Alphafold2 predicted structure of full length human VPS13B, orange indicates the rod consisting of 13 RBG domains. **(C)** Alphafold2 prediction of the Jelly Roll domain of VPS13B alone on the left and overlayed with the Jelly Roll Fold of anaphase promoting complex subunit Doc1p/Apc10 on the right. **(D)** Alphafold2 prediction of VPS13B internally tagged with Halo tag (magenta). **(E)** COS7 cells expressing VPS13B^Halo and kept at 37°C (top panel) or shifted to 20°C for 30 minutes before fixation (bottom panel) and then stained with Lipid Tox. Scale bar = 5 µm.

Supplemental Figure 2. Human FAM177A1 and FAM177B localize at the Golgi complex

(A) Bioplex data showing predicted partners of VPS13B. (https://bioplex.hms.harvard.edu/explorer/network.php). **(B)** HeLa cells expressing FAM177A1-GFP immunolabeled with anti-GFP, anti-GM130 and anti-TGN46 antibodies. **(C)** COS7 cells expressing FAM177A1-helix2-GFP (top) or FAM177A1-Hairpin-GFP (bottom). **(D)** Left panel, Sequence alignment of FAM177A1 and FAM177B. Right panel, Alphafold2 predicted structure of FAM177A1 and FAM177B. **(E)** HeLa cells expressing FAM177B-flag fixed and immunolabeled with anti-flag and anti-GM130 antibodies. Scale bar = 10 µm. **(F)** HeLa cells expressing FAM177A1-GFP and Rab6T27N-RFP. Scale bar = 10 µm. **(G)** HeLa cells expressing GalT-RFP and VPS13B^Halo before (left panel) and after BFA treatment (5 µg/mL for 40 minutes, right panel). Scale bar = 10 µm.

Supplemental Figure 3. Generation of VPS13B and FAM177A1 KO HeLa cells and Zebrafish genome encodes and expresses a VPS13B homolog

(A) Sanger sequencing of *VPS13BKO*^{1} and *VPS13BKO*^{2} HeLa cells; superscripts indicate different clones. **(B)** *VPS13BKO*^{2} HeLa cells expressing FAM177A1-GFP **(C)** Sanger sequencing of *FAM177A1KO* homozygous HeLa cells **(D)** *FAM177A1KO* cells overexpressing VPS13B^Scarlet. Scale bar = 10 µm. **(E)** Side-by-side comparison of Alphafold2 predicted structrure of zebrafish Vps13b and human VPS13B. **(F)** Conserved syntenies of *VPS13B* in human and zebrafish validate orthology implied by sequence comparisons. A small part of human (*Homo sapiens*) chromosome 8 (Hsa8, green part in insert) has conserved synteny with a short portion of zebrafish (*Danio rerio*) chromosome16 near its right tip (Dre16, green portion in insert). **(G)** qRT PCR analysis of WT *vps13b* in early zebrafish embryos at 2 cell stage, 6 hpf, 24 hpf. **(H)** Genotyping of *vps13b* CRISPR target locus in WT zebrafish embryos injected with *vps13b* gRNAs/Cas9 and treated with 0.8 µg/mL BFA (samples 1-6), *fam177a1a;fam177a1b DKO* embryos injected with *vps13b* gRNAs/Cas9 and treated with 0.8 µg/mL BFA (samples 7-12), WT zebrafish embryos injected with *vps13b* gRNAs/Cas9 and treated with DMSO (samples 13-18), WT zebrafish embryos treated with DMSO (samples 19,20), *fam177a1a;fam177a1b DKO* embryos injected with *vps13b* gRNAs/Cas9 and treated with DMSO (samples 21,22).

bioRxiv preprint doi: https://doi.org/10.1101/2023.12.18.572081; this version posted December 18, 2023. The copyright holder for this preprint (which was not certified by peer review) is the author/funder, who has granted bioRxiv a license to display the preprint in perpetuity. It is made available under aCC-BY 4.0 International license.

Fig. S1

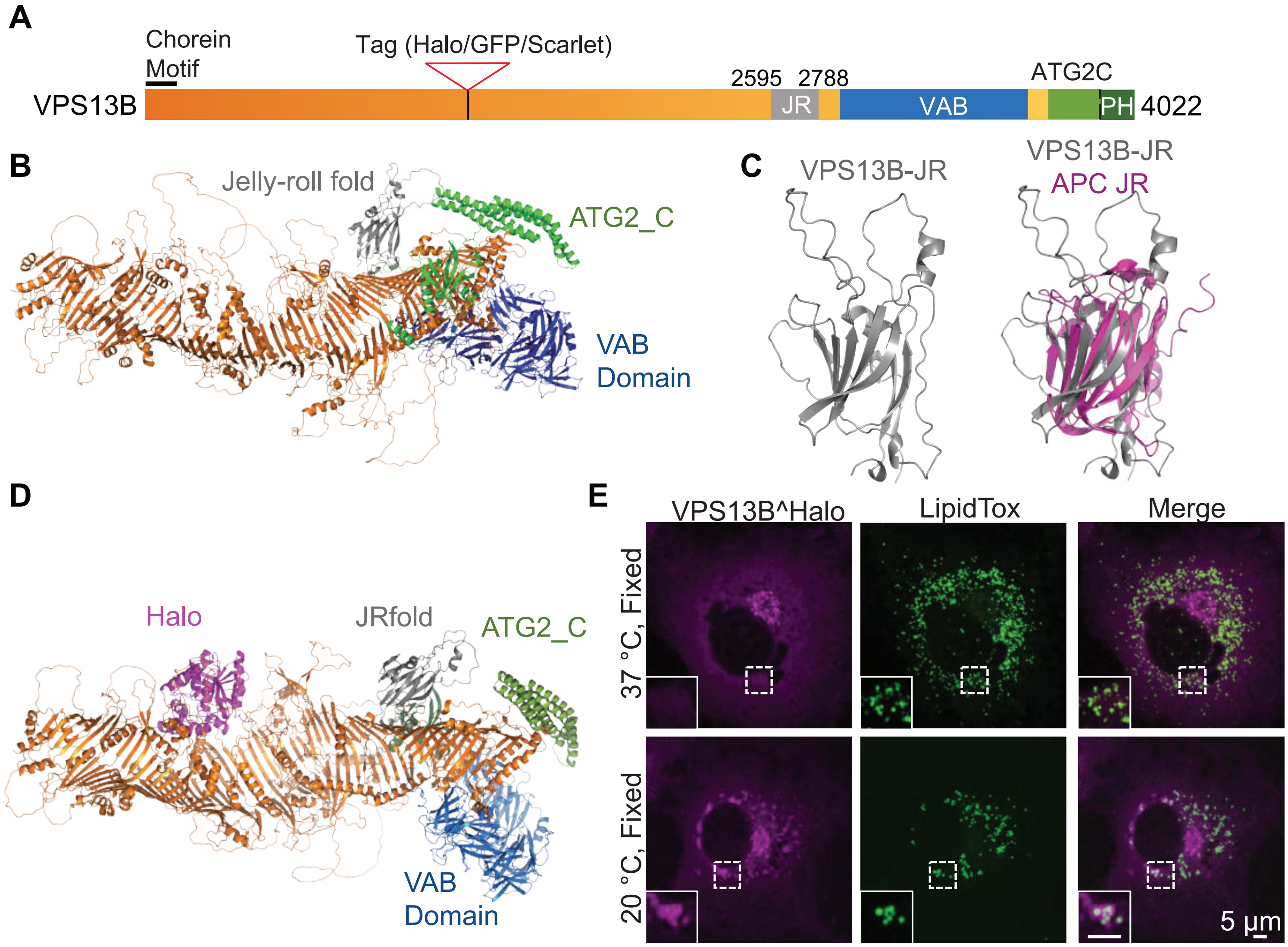

bioRxiv preprint doi: https://doi.org/10.1101/2023.12.18.572081; this version posted December 18, 2023. The copyright holder for this preprint (which was not certified by peer review) is the author/funder, who has granted bioRxiv a license to display the preprint in perpetuity. It is made available under aCC-BY 4.0 International license.

Fig. S2

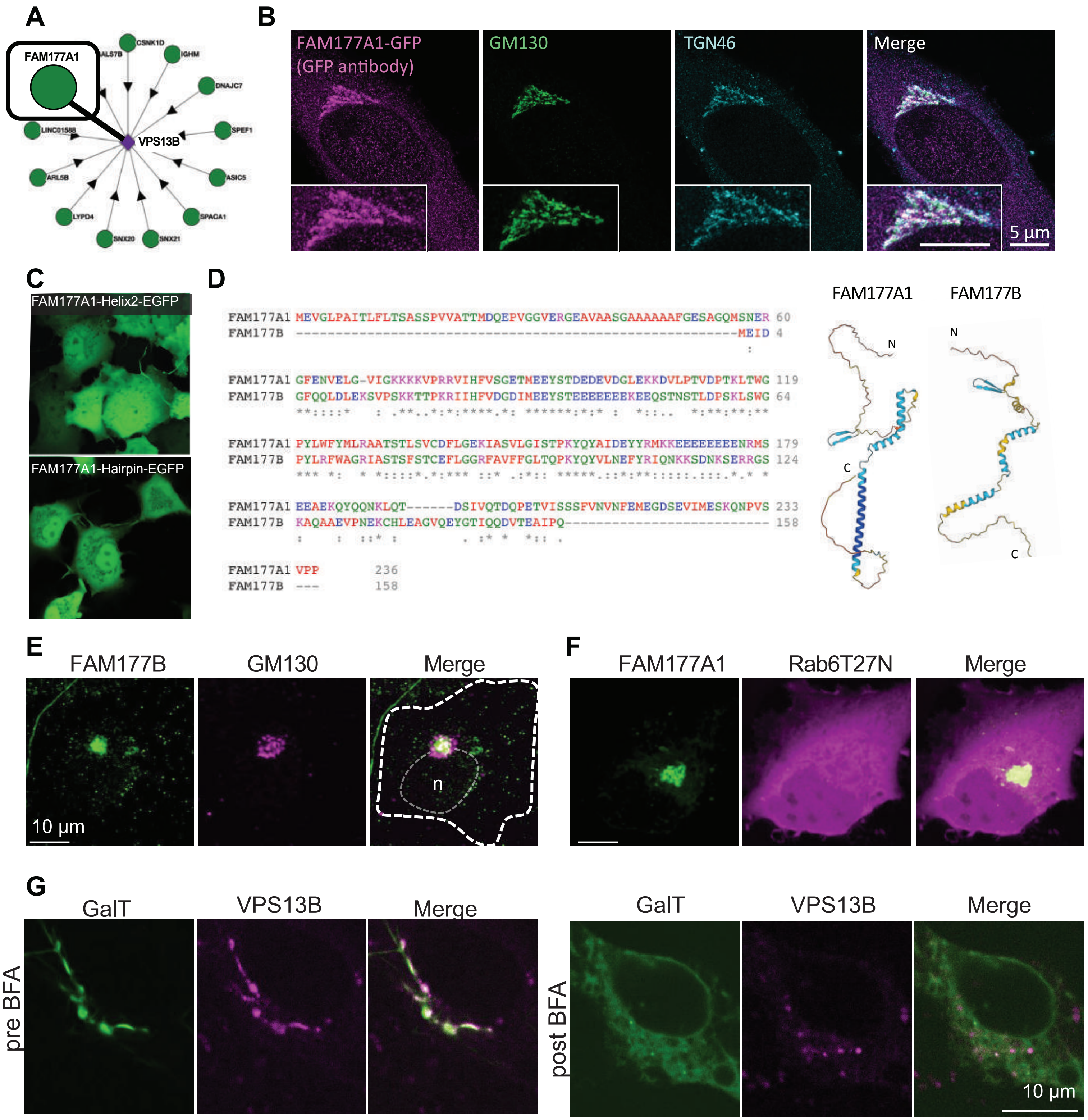

bioRxiv preprint doi: https://doi.org/10.1101/2023.12.18.572081; this version posted December 18, 2023. The copyright holder for this preprint (which was not certified by peer review) is the author/funder, who has granted bioRxiv a license to display the preprint in perpetuity. It is made available under aCC-BY 4.0 International license.

Fig S3

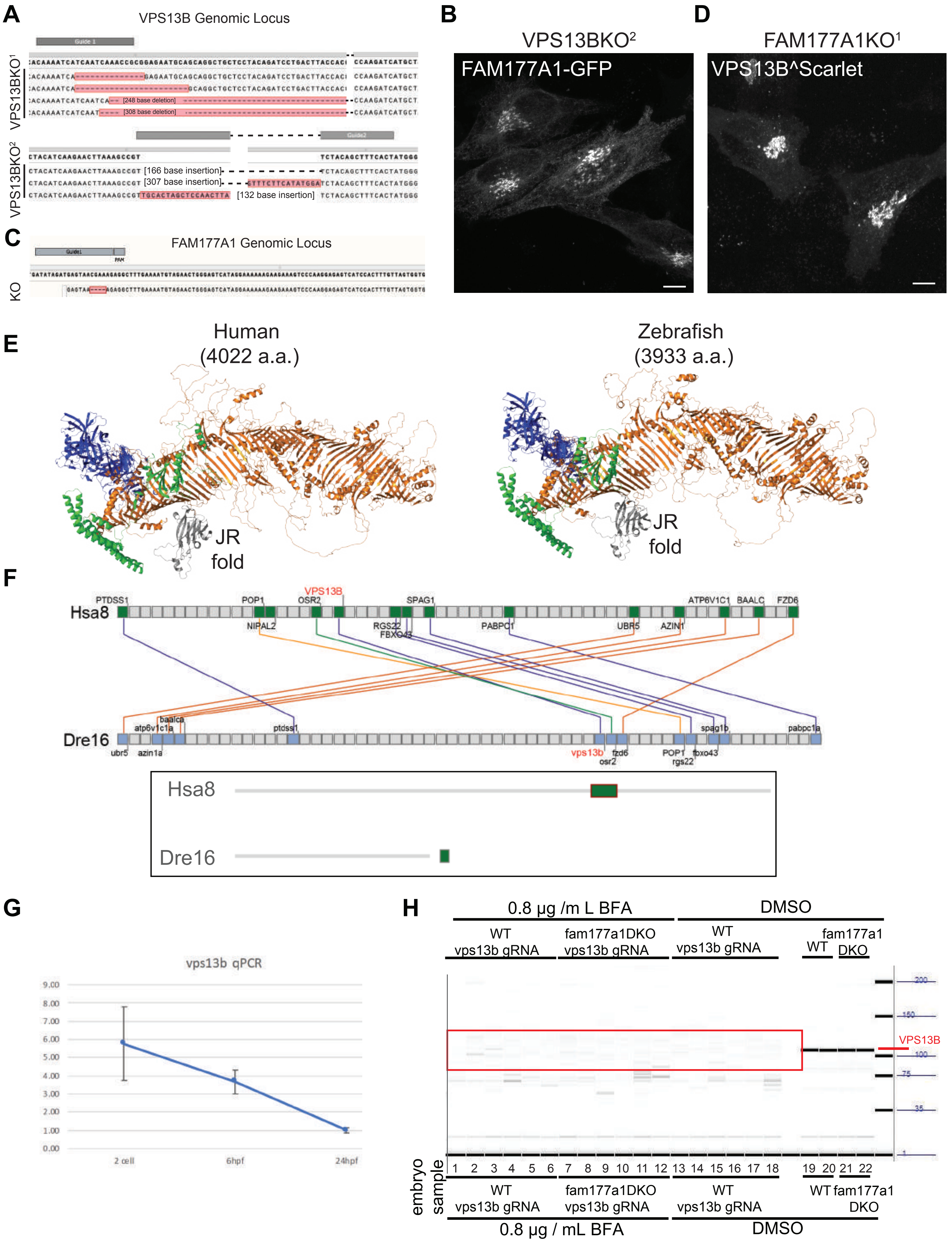

bioRxiv preprint doi: https://doi.org/10.1101/2023.12.18.572081; this version posted December 18, 2023. The copyright holder for this preprint (which was not certified by peer review) is the author/funder, who has granted bioRxiv a license to display the preprint in perpetuity. It is made available under aCC-BY 4.0 International license.

Supplemental Tables

Table S1. List of primers used in this study.

VPS13B Guide 1 sequencing forward	CAAATGGGGAAAAATTAAGAACC
VPS13B Guide 1 sequencing reverse	ACTTCCAGCCTGGGGATATT
VPS13B Guide 2 sequencing forward	TTCGGCCTGAGATAACGAAC
VPS13B Guide 2 sequencing reverse	TGTAAAATAAAGCCCCCTTGTT
FAM177A1 sequencing forward	CACCGATATAGATGAGTAACGAAA
FAM177A1 sequencing reverse	AAACCTTTCGTTACTCATCTATAT
FAM177A1 Fragment1 Halo Forward	CTCGAGATTTCCGGCtgataaacccgctgatcagc
FAM177A1 Fragment1 Halo Reverse	CATTCCACTTCCTCCtggtgggacagagactggat
FAM177A1 Fragment2 Halo Forward	ccaGGAGGAAGTGGAATGGCAGAAATCGGTACTGGCTTT
FAM177A1 Fragment2 Halo Reverse	ttatcaGCCGGAAATCTCGAGCGTCGACA
*FAM177A1*_Fragment1 Snap Forward	CTGGGTtgataaacccgctgatcagcctc
FAM177A1 Fragment1 Snap Reverse	CATTCCACTTCCTCCtggtgggacagagactggat
FAM177A1 Fragment2 Snap Forward	ccaGGAGGAAGTGGAATGGACAAAGACTGCGAAATGA
*FAM177A1*_Fragment2_Snap reverse	atcagcgggtttatcaACCCAGCCCAGGCTTG
**Zebrafish study**	
**Primers used in the qPCR and genotyping**	**Sequence**
vps13b qPCR forward	CCGTCAGACACCACAGATCT
vps13b qPCR reverse	AGATTCACTGCAATGCCCTGA
rpl13a qPCR forward	TCTGGAGGACTGTAAGAGGTATGC
rpl13a qPCR reverse	AGACGCACAATCTTGAGAGCAG
*vps13b*_geno_F	GTTCCTGCCCTCCGACACAC
*vps13b*_geno_R	GCAGACACACCACCAGCGT

Table S2. FLASH-PAINT Nanobody Sequences

**Target**	**Nanobody**	**Sequence (3’ → 5’)**
VSP13b-GFP	Anti-GFP-Nanobody-A3	NB - TT TCTTCATTAGCG
TGN46	Anti-Rabbit-Nanobody-A15	NB - TT ATAGTGATTGGA
GM130	Anti-Rabbit-Nanobody- A39	NB - TT TTATGTTCTGCT
GOLGA1_1 Golgin-97	Anti-Rabbit-Nanobody-A8	NB - TT ATGTTAATGGGT

bioRxiv preprint doi: https://doi.org/10.1101/2023.12.18.572081; this version posted December 18, 2023. The copyright holder for this preprint (which was not certified by peer review) is the author/funder, who has granted bioRxiv a license to display the preprint in perpetuity. It is made available under aCC-BY 4.0 International license.

GRASP65	Anti-Rabbit-Nanobody-A38	NB - TT ATTTAGTGTAGC
GOLGB1 Giantin	Anti-Rabbit-Nanobody-A20	NB - TT ATATGATCTCCG
COPI (CMIA10)	Anti-Mouse-Nanobody-A27	NB - TT AAAAAGTTCGAG

Table S3. FLASH-PAINT Imager Sequence

**Imager name**	**Sequence**	**3'-mod**
R2-6nt	TGGTGG	Cy3B

Table S4. FLASH-PAINT Adapter Sequences

**Adapter Name**	**Sequence (3’ → 5’)**
A3-5xR2	ACCACCACCACCACCACCA AA CGCTAATGAA
A15-5xR2	ACCACCACCACCACCACCA AA TCCAATCACT
A39-5xR2	ACCACCACCACCACCACCA AA AGCAGAACAT
A8-5xR2	ACCACCACCACCACCACCA AA ACCCATTAAC
A38-5xR2	ACCACCACCACCACCACCA AA GCTACACTAA
A20-5xR2	ACCACCACCACCACCACCA AA CGGAGATCAT
A27-5xR2	ACCACCACCACCACCACCA AA CTCGAACTTT

Table S5. FLASH-PAINT Eraser Sequences

**Eraser Name**	**Sequence (3’ → 5’)**
E3-5xR2	TTCATTAGCG TT TGG
E15-5xR2	AGTGATTGGA TT TGG
E39-5xR2	ATGTTCTGCT TT TGG
E8-5xR2	GTTAATGGGT TT TGG
E38-5xR2	TTAGTGTAGC TT TGG
E20-5xR2	ATGATCTCCG TT TGG
E27-5xR2	AAAGTTCGAG TT TGG

bioRxiv preprint doi: https://doi.org/10.1101/2023.12.18.572081; this version posted December 18, 2023. The copyright holder for this preprint (which was not certified by peer review) is the author/funder, who has granted bioRxiv a license to display the preprint in perpetuity. It is made available under aCC-BY 4.0 International license.

Table S6. FLASH-PAINT Adapter and Imager Concentrations

**# Round**	Protein	Adapter – Concentration	Imager – Concentration
Round 1	VPS13b	A3-5xR2 – 20 nM	R2 – 200pM
Round 2	GM130	A15-5xR2 – 20 nM	R2 – 500 pM
Round 3	TGN46	A39-5xR2 – 20 nM	R2 – 200 pM
Round 4	COPI	A8-5xR2 – 20 nM	R2 – 500 pM
Round 5	Golgin97	A38-5xR2 – 20 nM	R2 – 500 pM
Round 6	Giantin	A20-5xR2 – 20 nM	R2 – 500 pM
Round 7	Grasp65	A27-5xR2 – 20 nM	R2 – 300 pM

bioRxiv preprint doi: https://doi.org/10.1101/2023.12.18.572081; this version posted December 18, 2023. The copyright holder for this preprint (which was not certified by peer review) is the author/funder, who has granted bioRxiv a license to display the preprint in perpetuity. It is made available under aCC-BY 4.0 International license.

Supplemental Sequences

>vsp13b predicted peptide sequence

MLESYVSPLLMSYVNRYIKNLKPSDLQLSLWGGDVVLSKLDLRLDVLEQELKLPFTFLSG

HIHELRIHVPWTKLSSEPVVVTINTMECILKLRDGATDDSESCASSSTSRSAPEGSRAAV

RARRQQQQQQGPSGPDLPPGYVQSLIRRVVNNVNIVVNNLILKYVEDDIVLSVNITSAEC

YTVDELWDRAFMDISAPELVLRKVINFSDCTVCLDRRNASGKIEFYQDPLLYKCSFRTRL

HFSYDSINAKIPAVIKIHTMVESLKLSLTDQQLPMFLRLLELGVALYYGEMEERREVERE

DSGPLRDSIPGLRDGQCDEDEDGDEDGEQGWVSWAWSFIPAMVSAEEEERERAEGEQGAE

FVECLEPGAPRPLSTAHRDPVVSIGFYCTKASVTFKLTESCSESSFYSPQKLKSREVLSV

EQEGITVEALMMGEPFFDCQVGVVGCRALCLKGIMGVRDFEDNMNRKEEDAVFFRCGDTL

SLKGMTYLTNSLFDYRSPENNGVRAEFILEGNLHKETYTEAAGLQRYGAFYMDYLYMMES

SSRVCAGLQDGAALAVRLQETSLKRVVFGALDLQLHSSAVHRMLKMITCTLEHEYQPYCR

AQPPDVVEECVSVDPEQVAALEECIPVRQTIVTLMRATVTIPAAEYNLLHLILPTLLGHK

VTSAQTSVPQFQLLRPLPALRLQFQRVTFEHSQPMHEEEVTRAASSLKQPSHTLLHHCYT

HCCLKVFELQAGLTVIGSEEASQPLIPIIPAFSTALYGKQLHLPAYWLRNPSVQVSECVL

ELPQVCVQATRAQVLLLHCMTRSWTHSEGGGACSGITDSLISHAYNTTGVKSCAPVLEVC

VQRVELKVCVRPALLCVSGTLGAVKVCARTPGVCEGQKDQLVPLLQGPSDTTDLHSSRWL

SGSRKPASLLSPDLLQLTVQLPQQEHTPNPGAVLLLSVQGIAVNLDPVLSSWLLFHPQRT

SGSRQTQQVSMAMKKRREDEASVGSAALTRQASNQNSDYTSSPVKTKTVTESRPLSIPVK

VFPSAEECPVSPEEQMKNLITHTWNAVKHLTLQVELQSCCVFLPSDTLPSPSTLVCGDVP

GTVRSWYHSQVCMPGTLVVCLPQISVLSAGHRRMEPLQDGPLTVPRPVLEEGGAFPWTVC

VRQLSVYSLLGHQRSLSVVDPLGCTSTLALTAPRLQPAARDAFIICLHVDLQPLQLQCSN

PQVQLVCALWCRWMQIFSVLERLQTRGAQRAAAGFPECSAPAAGPASPVHSSAGTAPPDT

STCSPSADLGTPTEADSAHTDDPAAGETLTLEQQTCSISGSSRRLSVWMQWMLPRLTLKL

FSSEPASRTTELCVLAEMEDLSASVDIQDVYTKVKCKVGSFHIDHYRSSAEEAAVVLSCT

EKLNRRTVLLRPLGRQEPHSAFSFFPPAAAKALEVSHQQHGFLSVTYTQAVTRNVRHKLT

ARQERMAATPRLSEDTSDASPQHLHEILLTAQPFDLVLSCPLLAAVARVFHLPPSLRPPT

RERISAQPMRGHALSSSSLPLVYVNTSVIRIFCPLEDTHGSTHSQSKHWKEDTVVLKIGS

VSVAPQADNPLPRSVLRKDIYQRALNLGVLRDPGSEVEDRQYQIDLQCMNIGTAQWAELQ

PEGAGPGGVTSADTERSSQNPALEWNMASSIRRQQEKRVILSPIITDFSVRITAAPAVIY

RKPLSADHGPAEEVVVCGHSLEMNITSNLELFLSAAQVQLLQRLLQDNMRLTEDADTTAD

VCGQQQQQAAAVSGCVESVCVGGAPCGQDSGFGSDSARLRIVQHRLARPPQQPTVTKSLS

bioRxiv preprint doi: https://doi.org/10.1101/2023.12.18.572081; this version posted December 18, 2023. The copyright holder for this preprint (which was not certified by peer review) is the author/funder, who has granted bioRxiv a license to display the preprint in perpetuity. It is made available under aCC-BY 4.0 International license.

FVPFDVFLTAGRISLMTYATAPTDPADPTAPASPPAPPAPAVQDGGLASLTADSLLRGGS

CMSAPGRSSARQALGVTVVRQPGRRGDTHTCLQPLLLLQVMQPSVLLSCQHRRQRLELSL

FDLSLKGPVADYRSHDAGKSLPESLDYSVFWLQTVAGEADGRTGIPPPLLSVSVRDFLSA

PELQVEVSRPLRVSPTPAKVEQAKLFWRTLFPEGEALPTDKTGPTPSCSEAPPTVDRLLG

ALQTPVPFRRLALHAVQLVLSVETQTCSSVTLSVSALTSTLTLQNNSSSRPADGFKEVCV

SLQCEDLLIRTALRERSSVFVGPFSCSADLEAHWCRHSGSSAPDAPGPPRVLIDMKGGLL

QVFWGQLQFNCLSEFLEQLQNYWSQLSTVEAEPQDKPCPSMPPPTSLSAQSEHSSDDLRT

GLFQYIQDSACQRLPAPHEVVFWRETEDSPGVMLWRYPEPRAITFLRITPVPFNTTEDPD

ISTADLGDVLQVPCSLEFWDELQRVFVPYREFSLSESSVCELTLPTLTPDTQQTDLVTSD

LWRMVLNSNSDGGDESSDSESGSQVHCEQLVCPTALAACTRVDSCFAQWCVSSVGVSMRL

AYLQLHFCHNLDQLGTVPCQKLRPFLPDRKLPQDQEFAVVCVREPCVFVRQWCGVVQSCV

ELSLSCALSCSLLEYRNLTLLPVLQPTRMQAHATHTLTHTHAQHTLHCHATLQPLHAAIG

QYAIHTLDRALQAWRQNGVSDAEEVVFCHYVICNDTQEVLRFGQVDTDENILLQSNQSHQ

YSWRTHRSPQLLHICMEGWGNWRWAEPFSVDDAGTLLRTIQHRGQTASLIIRVTQLSAVQ

KQVVISGRQVVCSFLHQPIELRLLQRVCVDGQPLLRARVCRLEPDCRPPAFVLEHAELTE

VCVRACGEDAWSQDVCLEHSDTHNSSVVQVPSSNGSLLHVWCTRVLLEPNTHTQQRVVVF

SPLFMMWSHLPEPVLVHVEKRSLGLRDTQLIPGRGHQEALLNAEADLTHHLTFQAREEEG

ASHCAVPVSTAVVKQILSRASAEHQQNPQNILEHFCGEKKPSRAAWPYSSREAERGAGEP

VAQWDSPMQVRLSVWRAGLNTLLVELLPWALLVNRSHCDLWLFEAENIIVQIPAGGTIVP

PNLTDAFQIGIYWPHTNTVHKSPAVQLVHEVSSPRWPEGGGADVLLLDEEGFIHTDITLG

TQPGNLKLCQFCVSSSVKFGIQVLQIEDKTVLVNNTTHTFKCRAVIPQHTAVTPAQLCPV

PESSVFALGPAGAAGDQVCCALPCWDVLRGPSSEDPPLGPQLLQLSCEGCVLRWSPAAPV

RSDRPRQSLPVPVEPSSDWPFSTRPLVLTCQEHLGVTYLTVSEDQSPRMLIHNHCPKSLL

LKENTRECVRWPVFPRVLPAHSSVHHELLHQASSFPECRQKETLPTLRLRILTDTSTGPT

DTPTHSTDTPTDPTDTPTTPGWTEAIDISSPGTQVVFLPGFGCLYIDVLQQSGSVTLTLA

AESSAAELITQHRPCRQMLSFRLLLDEASVALCDDITSPSGSVELLRLTISKLLLLLPPC

EPPADPSTAPALHTLQVLCGGLQVDNQLYERASFHFPVMVCQEPQGEAELCPQQLQQFCA

ACFLSLSISVCADGQLDRLALRIRPARLYLEDTFIYYMKTLFHTYIPECAAGGRGCVRGV

ESAVPQEVLESMRALVSPLRLQKLSIEPLQLLVSIHASLKLYIASDHTPLAFSEFERGPV

CTTARHLVHTLAMHYAAGALFRAGWVVGSLEILGSPASLVRSIGNGVSDFFRLPYEGLTR

GPGAFISGVSRGTNSFIKHISKGTLTSITNLATSLARNMDRLSLDDEHYTRQEEWRRQLP

ETLGDGLRQGLSRLGISLLGAVAGIVDQPMQTFTRTLELPNSASSAARGVISGVGKGIVG

bioRxiv preprint doi: https://doi.org/10.1101/2023.12.18.572081; this version posted December 18, 2023. The copyright holder for this preprint (which was not certified by peer review) is the author/funder, who has granted bioRxiv a license to display the preprint in perpetuity. It is made available under aCC-BY 4.0 International license.

VFTKPIGGAAELVSQTGYGLLHGAGLWQLPKQLHQPTDNRSADAANSSAKYIWKMLQSLG

RAELHMALDVCMVSGSGQERAGCLLLSAEVLFVVSVCEDAQQQAFPITEIQCEHDTHTPG

RITLTLQQQRVNTDAEVEGVRERLSELQYTRLLDFVRGASPFLPAALQPSVSPAEPQRSV

CRTYQYQADAAFARVFVCRFQMVKNRALRIGFH

>vsp13b predicted cdna sequence

TGACGCGTGGACGCAGCCTGTGTGTGCGGAAGTAATGGTCGTGGTGTGTGTTTGTGTATG

TGGAGCAGCCGGACGCGTCGCATGAACCCGCGTGAGGGTGTGTCAGGATGTTGGAGTCGT

ATGTGTCTCCTCTGCTGATGAGTTATGTGAACCGCTACATAAAGAACCTGAAGCCGTCAG

ACCTGCAGCTGTCACTATGGGGCGGCGACGTGGTGCTGAGCAAACTGGACCTGCGGCTGG

ACGTGCTGGAGCAGGAGCTGAAGCTGCCCTTCACCTTCCTGAGCGGCCACATCCACGAGC

TGCGCATCCACGTGCCCTGGACCAAGCTGAGCTCAGAGCCGGTGGTCGTCACCATCAACA

CCATGGAGTGCATCCTGAAGCTGCGGGACGGAGCCACGGATGACTCTGAGAGCTGTGCCT

CCAGCTCCACCAGTCGCAGTGCTCCTGAGGGCTCCAGAGCTGCAGTGAGGGCCCGGCGAC

AGCAGCAGCAGCAGCAGGGCCCCAGCGGCCCCGACCTGCCCCCAGGCTATGTGCAGAGTC

TGATCCGGCGTGTGGTCAATAATGTGAATATCGTGGTCAATAACCTGATCCTGAAGTACG

TGGAGGACGACATCGTGCTGTCGGTGAACATCACGTCTGCGGAGTGTTACACGGTGGACG

AGCTGTGGGACCGGGCCTTCATGGACATCAGCGCTCCAGAACTGGTCCTCCGGAAGGTGA

TCAACTTCTCGGACTGCACCGTGTGTTTGGACCGCCGGAACGCCAGCGGGAAGATCGAGT

TTTACCAGGACCCGCTGCTCTACAAGTGCTCCTTCAGGACCCGTCTGCACTTCAGCTACG

ACAGCATCAACGCTAAGATCCCCGCCGTCATTAAAATTCACACTATGGTGGAGAGTCTGA

AGCTCTCCCTGACGGATCAGCAGCTGCCCATGTTCCTCCGGCTGCTGGAGCTGGGTGTAG

CGCTGTATTATGGGGAGATGGAGGAGCGGCGGGAGGTGGAGCGGGAAGACAGCGGCCCAC

TGCGAGACAGCATTCCCGGGCTGCGGGATGGGCAGTGTGATGAGGATGAGGATGGGGATG

AGGATGGGGAGCAGGGCTGGGTGTCGTGGGCCTGGTCCTTCATCCCAGCCATGGTGAGCG

CTGAGGAAGAGGAGCGCGAGCGTGCGGAGGGTGAGCAGGGTGCAGAGTTTGTGGAGTGTT

TGGAGCCCGGAGCCCCGCGGCCCCTCAGCACAGCACACAGAGACCCCGTAGTGTCCATCG

GCTTCTACTGCACTAAAGCCTCCGTCACCTTCAAGCTGACGGAGAGCTGTTCTGAGAGCA

GTTTCTACAGTCCGCAGAAGCTGAAGTCTCGAGAGGTGCTGAGCGTGGAGCAGGAGGGCA

TCACTGTGGAGGCGCTGATGATGGGCGAGCCGTTCTTCGACTGTCAGGTGGGCGTGGTGG

GCTGCAGAGCGCTGTGTCTGAAGGGCATCATGGGTGTGCGAGACTTTGAAGACAACATGA

ACAGAAAGGAGGAGGATGCTGTGTTCTTCCGCTGCGGTGACACACTGAGTCTGAAGGGCA

bioRxiv preprint doi: https://doi.org/10.1101/2023.12.18.572081; this version posted December 18, 2023. The copyright holder for this preprint (which was not certified by peer review) is the author/funder, who has granted bioRxiv a license to display the preprint in perpetuity. It is made available under aCC-BY 4.0 International license.

TGACGTATCTGACCAACTCACTGTTCGACTATCGGAGTCCAGAGAACAACGGAGTCCGCG

CAGAGTTCATCCTGGAGGGAAACCTGCACAAGGAGACGTACACAGAGGCTGCAGGTCTGC

AGAGATACGGAGCGTTCTACATGGATTACCTGTACATGATGGAGAGCAGCAGCAGAGTGT

GTGCGGGTCTGCAGGATGGCGCAGCTCTGGCGGTGCGGCTGCAGGAGACGTCGCTCAAGC

GGGTTGTGTTCGGGGCTCTGGATCTGCAGCTCCACAGCAGCGCCGTACACCGCATGCTGA

AGATGATCACATGCACACTGGAGCACGAGTACCAGCCCTACTGCAGAGCGCAGCCGCCCG

ATGTGGTGGAGGAGTGTGTGTCTGTGGACCCTGAGCAGGTTGCAGCGCTGGAGGAGTGTA

TTCCCGTGCGTCAGACCATCGTGACTCTGATGAGGGCGACGGTCACCATACCTGCAGCCG

AGTACAACCTGCTGCACCTCATCCTGCCCACACTACTGGGACACAAGGTCACCTCTGCGC

AGACGTCAGTCCCTCAGTTCCAGCTGCTGCGTCCTCTGCCTGCGCTGCGGCTGCAGTTCC

AGCGCGTCACGTTCGAGCACTCGCAGCCGATGCACGAGGAGGAAGTGACACGCGCAGCCA

GCAGCCTCAAACAGCCCTCACACACACTGCTGCACCACTGCTACACACACTGCTGCCTTA

AGGTCTTTGAGCTCCAGGCGGGTCTGACTGTGATTGGCTCAGAGGAGGCGTCGCAGCCGC

TCATACCCATAATTCCTGCTTTCAGTACTGCTCTCTATGGGAAGCAGCTCCACCTGCCTG

CGTACTGGCTCAGGAATCCGTCAGTGCAGGTCTCCGAGTGTGTGTTGGAGCTGCCGCAGG

TGTGTGTTCAGGCCACGCGCGCGCAGGTGCTGCTGCTGCACTGCATGACACGCAGCTGGA

CACACAGTGAGGGGGGCGGAGCCTGCAGCGGCATCACTGACAGTCTCATTAGCCACGCCT

ACAACACTACAGGTGTGAAGTCGTGCGCTCCTGTGCTGGAGGTGTGTGTTCAGCGTGTGG

AGCTGAAGGTGTGTGTGCGGCCGGCGCTGCTGTGTGTGTCTGGAACTCTGGGAGCCGTGA

AAGTGTGTGCCAGAACGCCCGGTGTGTGTGAAGGGCAGAAGGATCAGCTGGTGCCGCTGC

TCCAGGGTCCGTCAGACACCACAGATCTGCACAGCAGCCGCTGGTTGAGCGGGAGCCGTA

AGCCTGCGTCTCTGCTGTCCCCCGACCTCCTGCAGCTCACTGTGCAGCTGCCGCAGCAGG

AACACACACCCAACCCCGGTGCTGTTCTGTTGCTCAGTGTTCAGGGCATTGCAGTGAATC

TGGATCCGGTTCTGTCGTCCTGGCTGCTGTTCCACCCGCAGAGAACCAGCGGCAGCAGAC

AGACACAGCAGGTCTCCATGGCGATGAAGAAGAGGAGGGAGGATGAAGCTTCAGTCGGCA

GCGCTGCACTGACCAGACAAGCCAGCAACCAGAACTCCGACTACACCAGCAGCCCAGTCA

AGACCAAAACTGTGACAGAGTCCAGGCCTCTCTCTATCCCAGTAAAGGTTTTTCCCTCGG

CTGAGGAATGTCCCGTGAGTCCAGAGGAGCAGATGAAGAACCTGATCACACACACCTGGA

ACGCCGTCAAACACCTCACACTACAGGTGGAGCTGCAGTCCTGCTGTGTGTTCCTGCCCT

CCGACACACTGCCGTCTCCCAGTACGCTGGTGTGTGGGGACGTCCCGGGCACGGTGCGCA

GCTGGTACCACAGTCAGGTGTGTATGCCGGGCACGCTGGTGGTGTGTCTGCCGCAGATCA

GTGTGTTGAGCGCAGGACACCGCCGCATGGAGCCGCTGCAGGACGGCCCGCTCACTGTGC

bioRxiv preprint doi: https://doi.org/10.1101/2023.12.18.572081; this version posted December 18, 2023. The copyright holder for this preprint (which was not certified by peer review) is the author/funder, who has granted bioRxiv a license to display the preprint in perpetuity. It is made available under aCC-BY 4.0 International license.

CCAGACCCGTGCTGGAGGAGGGCGGTGCGTTCCCCTGGACGGTGTGTGTGCGTCAGCTGA

GTGTGTATTCTCTGCTGGGTCATCAGCGCTCTCTCAGTGTTGTGGATCCTCTGGGCTGCA

CCTCCACACTGGCCCTCACTGCACCCCGACTGCAGCCGGCCGCCCGAGACGCCTTCATCA

TCTGCCTGCATGTGGACCTGCAGCCGCTGCAGCTGCAGTGCTCCAACCCACAGGTCCAGC

TGGTGTGTGCTCTGTGGTGCCGCTGGATGCAGATCTTCAGTGTGTTGGAGCGTCTGCAGA

CTCGAGGAGCTCAGAGAGCGGCTGCGGGTTTCCCAGAATGCTCTGCTCCAGCCGCTGGCC

CCGCCTCCCCTGTGCACAGCAGCGCCGGCACCGCCCCGCCCGACACCAGCACCTGCAGCC

CGTCTGCTGACCTGGGCACGCCCACTGAGGCTGACTCTGCGCACACAGACGACCCCGCGG

CCGGCGAGACCCTGACCCTGGAGCAGCAGACCTGCAGCATCAGCGGCTCCAGCAGGAGAC

TCAGCGTCTGGATGCAGTGGATGCTGCCGAGACTCACACTCAAGCTGTTCTCCAGCGAGC

CCGCGAGCAGAACCACCGAGCTGTGTGTGCTGGCAGAGATGGAGGACCTGAGCGCGTCTG

TGGACATTCAGGACGTTTATACCAAGGTGAAGTGCAAAGTGGGAAGCTTCCATATAGATC

ACTACAGAAGCAGTGCTGAAGAGGCTGCGGTCGTCCTCTCTTGCACTGAAAAGCTAAACA

GACGCACGGTTCTGCTGCGGCCGCTCGGCAGACAGGAGCCGCACAGCGCTTTCAGCTTCT

TTCCCCCTGCGGCAGCGAAGGCTCTGGAGGTCTCGCACCAGCAGCACGGCTTCCTGTCGG

TGACCTACACGCAGGCAGTGACGCGGAACGTGCGGCACAAGCTGACGGCACGACAGGAGC

GCATGGCGGCGACCCCCAGGCTGAGCGAGGACACGAGCGACGCTTCACCACAGCACCTGC

ACGAGATCCTGCTGACGGCCCAGCCCTTCGACCTGGTGCTGTCCTGCCCTCTGCTGGCGG

CAGTGGCACGCGTCTTCCACCTGCCCCCATCACTGCGCCCACCCACCCGCGAGCGCATCT

CTGCTCAGCCAATGAGAGGACACGCACTGTCTTCCAGCAGCCTGCCGCTGGTCTACGTCA

ACACCAGTGTGATCCGCATATTCTGCCCCCTGGAGGACACACACGGCAGCACACATTCAC

AGTCCAAGCACTGGAAGGAGGACACAGTTGTGCTGAAGATTGGATCAGTGAGCGTCGCTC

CTCAGGCTGATAATCCACTGCCGCGCTCCGTCCTGCGCAAAGACATCTACCAACGGGCGC

TGAATCTGGGCGTGCTGCGTGACCCGGGCTCTGAGGTGGAGGACCGCCAGTATCAGATCG

ACCTGCAGTGCATGAACATAGGGACGGCGCAGTGGGCGGAGCTACAGCCAGAGGGGGCAG

GGCCAGGCGGTGTCACATCGGCGGATACAGAGAGGAGCTCACAGAACCCTGCACTGGAGT

GGAACATGGCCAGCAGTATCCGCCGGCAGCAGGAGAAGCGAGTGATCCTCTCTCCGATCA

TCACAGATTTCTCTGTGCGCATCACAGCAGCTCCGGCCGTCATCTACCGCAAGCCCCTCT

CAGCAGATCACGGCCCAGCGGAGGAGGTGGTGGTGTGTGGTCACAGTCTGGAGATGAACA

TCACGTCTAACCTGGAGCTGTTCCTGAGCGCAGCACAAGTGCAGTTACTGCAGCGCCTCC

TGCAGGACAACATGAGGCTGACGGAGGACGCAGACACCACTGCAGACGTGTGTGGTCAGC

AGCAGCAGCAGGCCGCAGCAGTGTCGGGCTGTGTGGAGAGTGTGTGTGTCGGTGGCGCCC

bioRxiv preprint doi: https://doi.org/10.1101/2023.12.18.572081; this version posted December 18, 2023. The copyright holder for this preprint (which was not certified by peer review) is the author/funder, who has granted bioRxiv a license to display the preprint in perpetuity. It is made available under aCC-BY 4.0 International license.

CCTGTGGGCAGGACAGTGGTTTCGGCAGTGACAGTGCCCGCCTGCGTATCGTCCAGCACC

GGCTCGCGCGCCCGCCACAGCAGCCCACCGTCACTAAGAGCCTGAGCTTCGTCCCGTTCG

ATGTGTTCCTGACGGCCGGCCGCATCTCCCTGATGACCTACGCCACGGCACCCACCGACC

CCGCTGACCCCACCGCGCCCGCTAGCCCCCCCGCTCCCCCCGCACCGGCAGTACAGGATG

GTGGTCTGGCCTCTCTGACGGCTGACAGTCTGCTGCGCGGTGGCTCCTGCATGTCAGCTC

CGGGCCGCAGCTCTGCGCGTCAGGCTCTGGGGGTGACGGTGGTGCGGCAGCCGGGCCGGC

GGGGGGACACACACACCTGTCTGCAGCCACTGCTGCTCCTGCAGGTGATGCAGCCATCCG

TTCTGCTGAGCTGCCAACACCGCCGGCAGCGGCTGGAGCTCTCACTGTTCGACCTCTCGC

TAAAGGGGCCCGTCGCTGACTACAGGAGCCACGATGCAGGTAAGTCTCTGCCGGAGTCGC

TGGACTACAGTGTGTTCTGGCTGCAGACGGTGGCAGGTGAGGCAGACGGGCGCACCGGGA

TCCCTCCGCCACTGCTGTCTGTGTCCGTCAGAGACTTCCTGAGCGCACCAGAGCTGCAGG

TGGAGGTCAGCCGGCCGCTGCGGGTCAGTCCCACACCTGCGAAGGTGGAGCAGGCCAAAC

TCTTCTGGAGGACACTGTTCCCTGAGGGGGAAGCCCTGCCCACAGACAAAACAGGCCCCA

CCCCCAGCTGCAGTGAAGCTCCGCCCACAGTGGACCGGCTGCTGGGTGCGCTGCAGACAC

CTGTGCCGTTCCGCAGGCTGGCACTGCATGCAGTGCAGCTGGTGCTGAGTGTGGAGACGC

AGACGTGCAGCAGTGTGACGCTCTCAGTGTCTGCCCTGACCAGCACACTCACACTGCAGA

ACAACAGCAGCAGCAGACCCGCCGACGGGTTTAAGGAGGTGTGTGTGTCGCTGCAGTGTG

AGGACCTGCTGATCCGCACGGCTCTGAGGGAGCGCAGCTCAGTGTTTGTGGGTCCGTTTT

CCTGCAGTGCGGATCTAGAAGCTCATTGGTGCAGACACAGTGGAAGCTCCGCCCCTGATG

CACCGGGACCGCCCAGAGTGTTGATTGACATGAAGGGGGGCCTGCTGCAGGTGTTTTGGG

GTCAGCTGCAGTTCAACTGTTTGTCTGAGTTTCTGGAGCAGCTGCAGAACTACTGGAGTC

AACTGAGCACTGTGGAGGCGGAGCCACAGGATAAGCCCTGCCCCTCAATGCCTCCACCCA

CCTCCCTCTCTGCCCAATCAGAACACTCGTCTGATGACCTGCGCACTGGGCTCTTCCAGT

ACATACAGGATTCAGCGTGCCAGCGGCTGCCAGCTCCTCATGAGGTGGTCTTCTGGAGGG

AGACTGAGGATTCTCCAGGTGTGATGCTGTGGCGTTACCCTGAGCCGCGCGCCATCACCT

TCCTCAGAATCACACCTGTGCCTTTCAACACCACTGAAGACCCCGACATCAGCACCGCAG

ACCTGGGGGACGTGCTGCAGGTCCCCTGTAGTCTGGAGTTCTGGGACGAGCTGCAGCGAG

TGTTTGTGCCTTACCGAGAGTTCAGCCTATCAGAGAGCAGCGTATGTGAGCTGACCCTGC

CCACTCTGACCCCCGACACACAGCAGACTGACCTGGTGACCTCTGACCTCTGGAGGATGG

TGCTCAACAGCAACAGTGATGGAGGAGATGAGAGCTCTGACAGTGAGTCGGGCTCTCAGG

TGCACTGTGAGCAGCTGGTGTGTCCGACCGCTCTGGCCGCGTGTACGCGTGTGGACTCGT

GTTTCGCGCAGTGGTGTGTGTCATCAGTCGGGGTCTCTATGCGTCTGGCGTACCTGCAGC

bioRxiv preprint doi: https://doi.org/10.1101/2023.12.18.572081; this version posted December 18, 2023. The copyright holder for this preprint (which was not certified by peer review) is the author/funder, who has granted bioRxiv a license to display the preprint in perpetuity. It is made available under aCC-BY 4.0 International license.

TGCACTTCTGCCACAACCTGGACCAGCTCGGCACAGTGCCATGTCAGAAGCTCCGCCCCT

TCCTCCCGGACAGGAAGCTGCCTCAGGATCAGGAGTTTGCGGTGGTGTGTGTGCGCGAGC

CGTGTGTGTTTGTGCGTCAGTGGTGTGGCGTTGTGCAGAGTTGTGTTGAGCTGAGCTTGT

CGTGTGCGCTGAGCTGCAGTCTGCTGGAGTACCGCAACCTCACACTGCTGCCCGTCCTGC

AGCCCACGCGCATGCAGGCCCACGCCACACACACACTCACACACACGCATGCGCAACACA

CACTGCACTGCCACGCCACGCTGCAGCCACTGCACGCCGCCATCGGACAGTACGCCATAC

ACACACTGGACCGAGCGCTGCAGGCCTGGAGACAGAACGGCGTGTCGGATGCAGAGGAAG

TAGTTTTCTGTCATTACGTGATCTGCAACGACACGCAGGAAGTGCTGCGCTTCGGACAGG

TGGACACCGATGAGAACATCCTGCTGCAGAGCAACCAGAGCCACCAGTACAGCTGGAGGA

CACACCGATCCCCACAGCTGCTGCACATCTGCATGGAGGGCTGGGGTAACTGGCGCTGGG

CTGAACCCTTCAGTGTGGACGATGCAGGAACACTGCTGAGAACCATCCAGCACCGAGGAC

AGACGGCGTCACTGATCATCAGAGTCACACAGCTGAGCGCAGTGCAGAAACAGGTGGTGA

TCAGTGGTCGGCAGGTGGTGTGTAGCTTCCTCCATCAGCCGATCGAGCTCCGCCTCCTGC

AGCGTGTGTGTGTGGACGGTCAGCCGCTGTTGCGTGCGCGTGTGTGTCGCCTGGAGCCCG

ACTGCAGACCGCCGGCGTTCGTGCTGGAGCACGCGGAGCTGACGGAGGTGTGTGTGCGCG

CGTGCGGAGAGGACGCCTGGTCGCAGGACGTGTGTCTGGAGCACAGCGACACACACAACA

GCTCAGTGGTGCAGGTGCCGTCTTCTAATGGGTCACTGCTGCATGTGTGGTGCACACGAG

TGCTGCTGGAGCCGAACACACACACACACAGCAGAGAGTGGTGGTGTTCAGTCCTCTCTTCA

TGATGTGGAGTCACCTGCCGGAGCCGGTGCTGGTGCACGTGGAGAAGCGCAGTCTGGGCC

TCAGAGACACACAGCTGATCCCCGGACGCGGACACCAGGAGGCGCTGCTGAACGCGGAGG

CAGACCTCACACACCACCTCACCTTTCAGGCCAGGGAGGAGGAGGGCGCATCTCACTGCG

CAGTTCCTGTGTCCACTGCTGTCGTCAAGCAGATCCTGAGCAGAGCATCAGCAGAACACC

AGCAGAACCCGCAGAACATCCTGGAGCACTTCTGTGGAGAGAAGAAGCCCAGCAGAGCAG

CCTGGCCATACAGCAGCCGAGAGGCAGAGCGGGGTGCGGGCGAGCCGGTGGCGCAGTGGG

ACAGCCCGATGCAGGTGCGTCTGAGTGTGTGGCGCGCGGGTCTGAACACACTGCTGGTGG

AGCTGCTGCCGTGGGCTCTGCTGGTCAACCGCTCACACTGCGACCTGTGGCTGTTCGAGG

CCGAGAACATCATCGTGCAGATCCCGGCCGGAGGAACCATCGTCCCGCCCAACCTCACGG

ATGCGTTCCAGATCGGCATTTACTGGCCGCACACTAACACAGTGCACAAGTCTCCTGCCG

TGCAATTGGTGCATGAAGTGTCGTCACCACGGTGGCCAGAAGGGGGCGGAGCTGATGTGC

TGCTGCTGGATGAGGAGGGATTCATCCACACGGACATCACACTCGGAACACAGCCAGGCA

ATCTCAAGCTCTGTCAGTTCTGTGTGTCGTCTTCAGTGAAGTTCGGGATTCAGGTTTTGC

AGATTGAGGATAAAACTGTCCTGGTGAACAACACGACACACACCTTCAAGTGTAGAGCTG

bioRxiv preprint doi: https://doi.org/10.1101/2023.12.18.572081; this version posted December 18, 2023. The copyright holder for this preprint (which was not certified by peer review) is the author/funder, who has granted bioRxiv a license to display the preprint in perpetuity. It is made available under aCC-BY 4.0 International license.

TGATTCCACAACACACAGCGGTCACACCTGCGCAGCTCTGTCCTGTCCCAGAATCCTCAG

TGTTTGCTCTGGGCCCCGCGGGTGCGGCTGGAGATCAGGTGTGCTGTGCTCTGCCCTGCT

GGGATGTGCTGCGGGGTCCGTCCTCAGAAGACCCTCCGCTGGGGCCGCAGCTCCTGCAGT

TGAGCTGTGAGGGGTGTGTGCTGCGGTGGAGCCCTGCGGCCCCGGTGCGCTCCGATCGGC

CACGACAGAGTCTTCCGGTGCCCGTAGAGCCCAGCTCCGACTGGCCCTTCAGCACCAGGC

CTCTGGTTCTGACCTGTCAGGAGCATCTGGGCGTCACGTATCTGACAGTGAGTGAAGATC

AGAGTCCACGCATGCTGATCCACAACCACTGCCCCAAGAGCCTCTTACTGAAGGAGAACA

CCAGAGAGTGTGTGCGGTGGCCGGTGTTCCCCCGTGTCCTCCCTGCGCACTCCTCCGTCC

ACCATGAGCTGCTCCATCAGGCCTCCAGCTTCCCTGAGTGCCGACAGAAGGAGACGCTTC

CCACCCTGCGGCTGCGCATCCTCACAGACACGTCCACAGGCCCCACAGACACACCCACAC

ATTCCACAGACACGCCCACAGACCCCACAGACACGCCCACTACGCCAGGGTGGACAGAAG

CCATTGACATCAGCAGTCCTGGAACACAGGTGGTTTTCCTCCCTGGTTTCGGCTGTTTGT

ACATTGATGTTCTTCAGCAGAGCGGCAGCGTCACCCTGACACTGGCAGCAGAGAGCAGCG

CAGCAGAGCTCATCACACAGCACAGGCCGTGCCGTCAGATGTTGTCCTTCCGGCTGCTGC

TGGATGAGGCCAGTGTTGCGCTCTGTGATGACATCACCAGCCCGTCCGGCTCCGTGGAGC

TGCTGCGCCTCACCATCTCCAAACTGCTGCTGCTGCTGCCCCCCTGTGAACCGCCCGCTG

ACCCTAGCACTGCACCTGCCCTCCACACGCTGCAGGTGCTCTGCGGCGGTCTGCAGGTGG

ACAACCAGCTGTATGAGCGTGCCAGCTTTCACTTCCCGGTCATGGTGTGCCAGGAGCCGC

AGGGCGAGGCGGAGCTGTGCCCACAGCAGCTGCAGCAGTTCTGCGCCGCATGCTTTCTGT

CCCTGAGCATCAGTGTGTGTGCAGACGGGCAGCTGGACCGCCTCGCCCTGCGCATCCGGC

CTGCCCGCCTCTACCTGGAGGACACCTTCATCTACTACATGAAGACGCTCTTCCACACCT

ACATTCCAGAGTGTGCAGCGGGGGGGCGGGGCTGTGTCAGGGGGGTGGAGTCAGCGGTGC

CGCAGGAGGTGCTGGAGTCGATGCGTGCTCTGGTGTCGCCGCTGCGGCTGCAGAAACTGT

CCATCGAGCCGCTGCAGCTGCTGGTCAGCATCCACGCCTCGCTGAAGCTCTACATCGCCT

CTGACCACACGCCGCTGGCCTTCTCTGAGTTCGAGCGCGGGCCCGTCTGCACCACCGCAC

GACACCTGGTGCACACACTCGCCATGCACTACGCAGCAGGAGCACTCTTCAGGGCAGGCT

GGGTTGTCGGCTCTCTGGAGATTCTGGGCAGTCCAGCCAGTCTGGTGCGTAGTATCGGGA

ACGGTGTGTCTGATTTCTTCCGTCTGCCGTATGAGGGTTTGACCCGCGGGCCAGGTGCGT

TCATCAGCGGCGTCTCCAGAGGAACAAACTCCTTCATCAAACACATCTCTAAAGGAACGC

TGACGTCCATCACGAATCTGGCCACGAGTCTGGCGCGTAACATGGACCGGCTGTCGCTGG

ACGATGAGCACTACACGCGGCAGGAGGAGTGGCGGCGGCAGCTGCCCGAAACACTGGGAG

ACGGACTGAGACAGGGGCTGTCCAGACTGGGCATCAGCCTGCTGGGAGCGGTCGCAGGAA

bioRxiv preprint doi: https://doi.org/10.1101/2023.12.18.572081; this version posted December 18, 2023. The copyright holder for this preprint (which was not certified by peer review) is the author/funder, who has granted bioRxiv a license to display the preprint in perpetuity. It is made available under aCC-BY 4.0 International license.

TCGTGGATCAGCCCATGCAGACGTTCACTCGTACGCTGGAGCTGCCAAACTCTGCGAGCA

GCGCGGCCCGCGGCGTCATCTCCGGCGTGGGGAAAGGCATTGTGGGAGTTTTTACTAAAC

CAATCGGAGGAGCCGCTGAGCTGGTGTCACAGACGGGCTACGGCCTCCTCCATGGCGCAG

GACTCTGGCAGCTCCCTAAACAGCTCCACCAGCCGACTGACAACCGATCCGCCGACGCCG

CTAACAGCAGCGCCAAATACATCTGGAAGATGCTGCAGTCTCTGGGCCGTGCGGAGCTCC

ACATGGCGCTGGATGTGTGTATGGTGAGCGGCTCGGGGCAGGAACGCGCCGGCTGTTTGC

TGCTGAGCGCTGAGGTGCTGTTTGTGGTCAGTGTGTGTGAGGACGCGCAGCAGCAGGCCT

TCCCCATCACCGAGATCCAGTGTGAACACGACACACACACACCTGGACGCATCACACTCA

CACTGCAGCAGCAGCGCGTCAACACTGATGCTGAGGTCGAGGGCGTCCGCGAGCGTCTGT

CGGAGCTGCAGTACACTCGGCTGCTGGATTTCGTGCGCGGTGCGTCTCCGTTTCTGCCCG

CGGCGCTGCAGCCGAGCGTCAGTCCAGCAGAACCGCAGCGCAGCGTCTGCAGAACATACC

AGTACCAGGCGGACGCCGCATTCGCACGCGTGTTCGTCTGCAGGTTCCAGATGGTGAAGA

ACAGAGCGCTGCGCATCGGCTTCCACTGACACACACACACACACACACACACACACACAC

ACTCTGCTGCAGCACACATGGCCTTCTGTGTGTGTGTGTCTATAATGATGCCAAAAGAGA

CGATCCACTGTTTATATGTAATTTCCTGATTAAATTTGTGAGAAAT
